# The influence of intraoral cryotherapy on postoperative pain and substance P in symptomatic apical periodontitis: randomized clinical study

**DOI:** 10.1038/s41598-024-64071-y

**Published:** 2024-06-17

**Authors:** Esraa Mohammed Hamza, Tarek Mustafa Abd El Aziz, Maram Farouk Obeid

**Affiliations:** Department of Endodontics, Faculty of Dentistry, Ain Shams University, 2 sixth of October St., Hadayek Al-Ahram-Haram, Giza, Cairo, Egypt

**Keywords:** Health care, Medical research

## Abstract

Cryotherapy is widely utilized in medicine, particularly for pain management. This randomized clinical trial aimed to assess the effect of intraoral cold pack application (cryotherapy) on postoperative pain (POP) and the level of Substance P (SP) in patients with symptomatic apical periodontitis (SAP). Enrolled patients were randomly assigned to either cryotherapy or control group. After adequate anesthesia, access cavity, and biomechanical preparation of the root canal system were completed, the first apical fluid (AF) sample (S1) was obtained. A custom-made intraoral ice-gel pack was applied for 30 min in the cryotherapy group, while no intervention was performed in the control group. The second AF sample (S2) was collected 30 min later in both groups. Patients were asked to complete the Visual Analogue Scale (VAS) questionnaire to assess their POP. Quantification of SP in AF samples was performed using the enzyme-linked immunosorbent assay (ELISA) test. Data were analyzed statistically, revealing a significant reduction in POP and SP levels in the cryotherapy group compared to the control group (P ≤ 0.05). Furthermore, a moderate positive correlation was observed between SP levels and POP (P ≤ 0.05). In conclusion, intraoral cryotherapy represents a simple and cost-effective option for controlling POP and reducing inflammation levels in patients with SAP.

## Introduction

Postoperative pain (POP) remains one of the common concerns for patients following endodontic treatment as it affects between 3 and 58% of patients^1^. As a result, its management is considered crucial for treatment success. Unfortunately, the presence and severity of preoperative pain, pulp and periradicular state, and the presence of periapical radiolucency all have an impact on POP^2^. Furthermore, our endodontic treatment may occasionally cause chemical, mechanical, or microbiologic damage to the periapical region, resulting in POP^3^.

It has been suggested that long-lasting anesthetic injection^4^ and occlusal reduction^5^ can be preventative strategies to lower POP. Additionally, a variety of medications are already available, such as nonsteroidal anti-inflammatory drugs, corticosteroids, and paracetamol, that serve to decrease POP^6^. Despite their wide use, it is important to acknowledge the potential for gastrointestinal intolerance^7^. As a result, implementing non-pharmacological interventions to maximize physical and psychological comfort and function has been adopted.

A recently applied therapeutic tactic is reducing tissue temperature. It is referred to as “cryotherapy”^8^. The name comes from the Greek words ‘cryos’ meaning ‘cold’ and ‘therapeia’ meaning ‘therapy’^8^. The notion of cryotherapy does not entail chilling the target tissue but rather transferring heat from a higher-temperature tissue to a lower-temperature subject^9^. Cryotherapy has been widely utilized in medicine, particularly for pain management in sports injuries^10^. It is also utilized in dentistry not only following oral surgeries and extractions but also in endodontics, where it is reported to be utilized after periradicular surgeries and, uniquely, during root canal treatments^12–17^. Several randomized controlled clinical trials have investigated cryotherapy treatment for pain relief following endodontic treatment since it is a low-cost, easy, and non-toxic procedure^12,17^.

The physiological tissue effects of cryotherapy can be categorized into three basic phenomena. The first is vascular, with early reflex vasoconstriction followed by cold-induced vasodilation (mediated by histamine-like substance release)^8^. This repetition reduces permeability, lowering tissue edema and swelling^8^. The second is neurologic since tissue cooling causes analgesia by reducing nerve conduction velocity^18^, and the last one is by slowing down the rate of metabolic processes, limiting the creation of free radicals in tissues, slowing down the rate of oxygen consumption, and preventing tissue hypoxia and further damage^19^. Even though the efficacy of cryotherapy has been proven in the literature, there is no standardization regarding the type, quantity, and temperature of the cryoagent used, as well as the method and duration of its administration^12–17^.

Multiple measures were employed to measure POP after endodontic treatment, including the Numerical Rating Scale (NRS), Verbal Rating/Descriptor Scale (VRS/VDS), and Visual Analogue Scale (VAS)^20,21^. Even though all are relatively reliable, VAS has been widely adopted due to its practicality^20^. Regrettably, these measures are heavily influenced by the patient's perception of pain^21^. Consequently, several studies have linked pain levels to the production of neuropeptides by sensory neurons, which are abundant in the pulp^21–24^. Substance P (SP) is one such neuropeptide that is produced by nociceptors and triggers neurogenic inflammation^22^. Previous research has found a correlation between elevated levels of SP in the pulp and neuron activity^22^. Furthermore, dynamic changes in SP expression within pulpal nerves have been observed following caries, which could affect inflammation and pain perception^22^. Therefore, SP may be a useful tool for assessing and comparing pain levels in patients who have undergone endodontic treatment^23,24^.

There has been no investigation into the therapeutic impact of intraoral cryotherapy on POP and SP levels, as per the previous findings. An earlier study, which used the same patient cohort, focused solely on the effect of intraoral cryotherapy on substance P levels in patients with symptomatic apical periodontitis (SAP) was published^25^. This current study builds on that work by assessing both postoperative pain (POP) in addition to substance P (SP) levels, providing a more comprehensive evaluation of the effects of intraoral cryotherapy. Additionally, this study explores the correlation between SP levels and pain scores, offering further insights into the relationship between biochemical markers and clinical symptoms. The current study aims to analyze, quantify, and correlate POP and SP levels in individuals with SAP who were treated with or without intraoral cryotherapy using the Visual Analogue Scale (VAS) and enzyme-linked immunosorbent assay (ELISA). The null hypothesis indicated that there is no significant difference in POP and SP levels between the experimental group receiving cryotherapy and the control group.

## Results

The study involved 20 patients who were randomly allocated equally to each tested group, with 10 cases in each group. No significant difference was found between the groups in terms of demographic data (as shown in Table 1) and preoperative pain levels (as shown in Table 2). All patients refrained from taking pain medication post-operatively, and there were no cases of loss to follow-up.

**Table 1:** Analysis and sample distribution of demographic data in both groups.

	Parameter	Control group	Cryotherapy group	P-value
Sex [n (%)]	Male	3 (30.0%)	2 (20.0%)	1
	Female	7 (70.0%)	8 (80.0%)	
Age (Mean ± SD) (years)		29.70 ± 6.29	25.15 ± 5.43	0.100

*Significant (P < 0.05).

**Table 2:** Comparison of preoperative pain levels using VAS in both groups.

Parameter	Control group	Cryotherapy group	P-value
Preoperative pain on percussion VAS (Mean ± SD)	9.20 ± 0.92	9.20 ± 0.79	0.968
Preoperative pain VAS (Mean ± SD)	9.30 ± 0.82	9.50 ± 0.85	0.518

*Significant (P < 0.05).

*Regarding POP* (Table 3), intergroup comparisons showed that the control group had a significantly higher mean value than the cryotherapy group at all intervals (P < 0.05) except after 7 days in which both groups had zero. Intragroup comparisons revealed a significant reduction of values at different intervals (P < 0.05).

**Table 3:** Comparisons of mean and standard deviation values for POP levels (VAS) between groups:

Intervals	Post-operative pain (VAS) (mean ± SD)		P-value
	Control group	Cryotherapy group	
6 h after	7.90 ± 1.10^Aa^	5.10 ± 2.38^Bb^	0.003*
24 h	5.50 ± 2.22^Ba^	2.10 ± 2.23^Cb^	0.004*
48 h	4.20 ± 2.25^Ba^	0.90 ± 1.52^Cb^	0.003*
72 h	2.30 ± 1.89^Ca^	0.10 ± 0.32^Db^	0.005*
7 days	0.00 ± 0.00^Da^	0.00 ± 0.00^Da^	0
P-value	< 0.001*	< 0.001*	

Different upper-case letters in the same column indicate statistically significant differences.

Different lowercase letters in the same row indicate statistically significant differences.

*Significant (*P* < 0.05).

*Regarding SP level*, both groups showed a significant reduction of substance P level after treatment (P < 0.05) (Table 4).

**Table 4:** Comparisons of substance P levels (pg/ml) within each group:

Intervals	Substance P level (pg/ml) (mean ± SD)		P-value
	Control group	Cryotherapy group	
S1	192.27 ± 32.94^Aa^	135.36 ± 66.86^Ab^	0.027*
S2	138.87 ± 35.18^Ba^	87.37 ± 53.79^Bb^	0.021*
S1–S2 (pg/ml) (mean ± SD)	53.40 ± 18.59^a^	48.00 ± 8.76^a^	0.518
P-value	0.003*	0.004*	

Different upper-case letters in the same column indicate statistically significant differences.

Different lowercase letters in the same row indicate statistically significant differences.

*Significant (*P* < 0.05).

**Figure 1:**
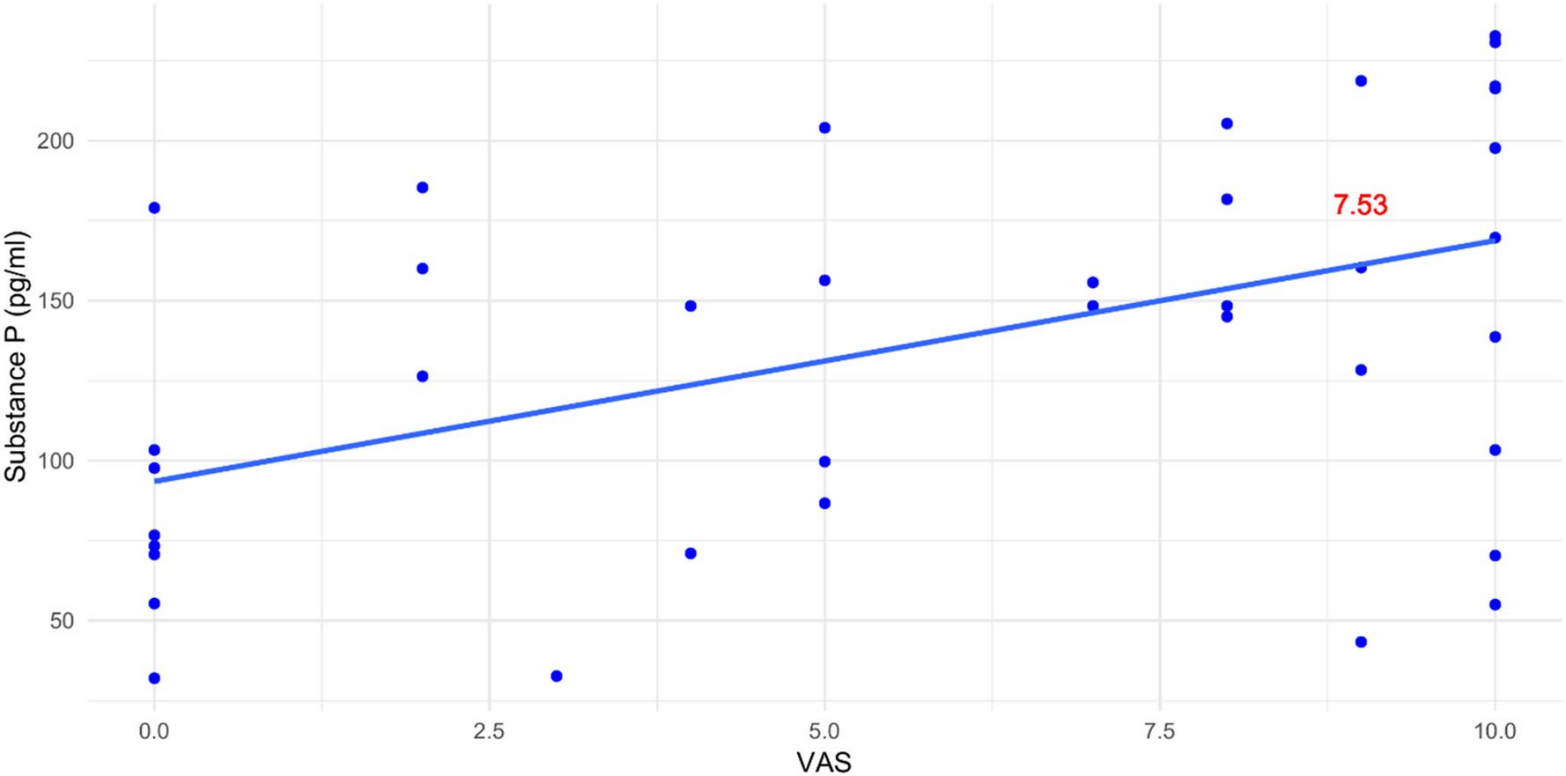
Scatter plot showing the correlation between SP level and POP within both groups. The slope value of the trend line is shown in red = 7.53 indicating a moderate positive correlation.

**Figure 2:**
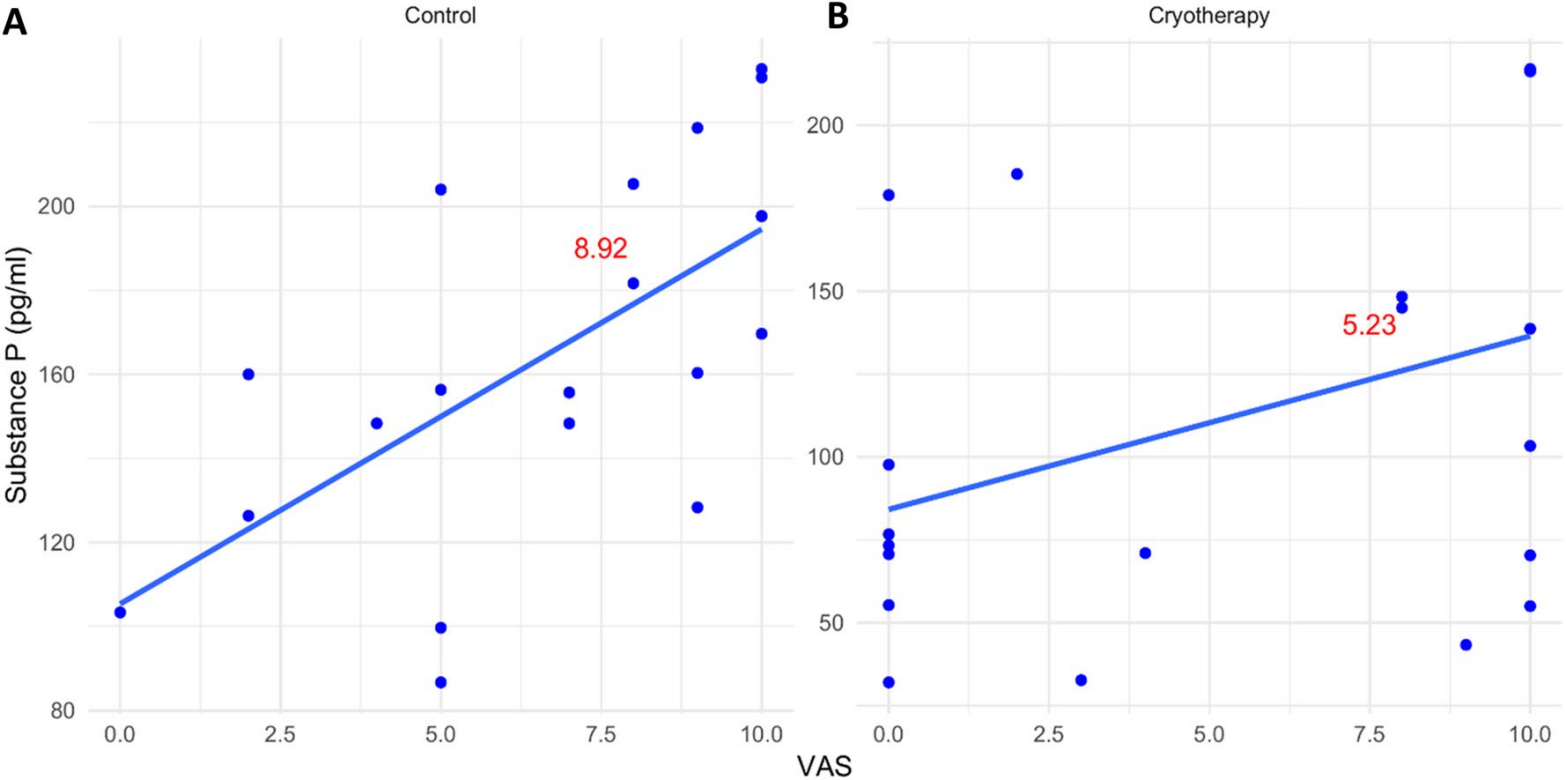
Scatter plot showing the correlations between SP level and POP within each group. The slope values of the trend lines are shown in red. (**A**) Control group (Slope = 8.92) indicating a strong positive correlation. (**B**) Cryotherapy group (Slope = 5.23) indicating a weak positive correlation.

There was an overall moderate positive correlation between SP and POP (Table 5, Fig. 1). For the control group, there was a statistically significant strong positive correlation, while for the cryotherapy group, the correlation was not statistically significant (Table 5, Fig. 2).

**Table 5:** Correlation between substance P level and POP.

Groups	Correlation coefficient	P-value
Control	0.671	0.001*
Cryotherapy	0.317	0.173
Overall	0.472	0.002*

*Significant (*P* < 0.05).

## Discussion

The purpose of this randomized controlled trial was to determine the effect of intraoral cryotherapy on the POP level and the expression of the neuropeptide SP. When compared to the control group, the results demonstrated that intraoral cryotherapy significantly lowered the level of POP and SP. As a result, the study’s null hypothesis was rejected.

We acknowledge that an earlier study involving the same patient cohort has been published^25^. The previous study evaluated the impact of intraoral cryotherapy on substance P levels in patients with symptomatic apical periodontitis. However, this current study extends the scope by including an assessment of postoperative pain (POP) and exploring the correlation between SP levels and POP. These additions provide a more detailed understanding of the therapeutic effects of cryotherapy, thus offering new insights beyond the earlier publication. Specifically, the current study correlates SP levels with pain scores, providing valuable information on the relationship between inflammatory mediators and clinical pain outcomes.

Pre-operative pain is one of the greatest predictors of post-operative pain^2^. As a result, having mandibular premolars with irreversible pulpitis and symptomatic apical periodontitis was a requirement for the patient’s inclusion to guarantee the presence of inflammation in and around the root. Pulp vitality was guaranteed first by pain sensation with ethyl chloride spray application then bleeding from the pulp during access cavity preparation (gold-standard)^26^. Apical periodontitis was confirmed by pain on percussion^12^.

Because only vital teeth were included in this study, the therapy was completed in one session (single visit) to prevent the use of intracanal medicines and rule out the presence of infected necrotic pulp.

Regarding cryotherapy and methods of cold application in Endodontics, many tactics were addressed in the literature starting from intracanal cryotherapy (i.e. using cold saline as a final irrigant)^13–16,27^, Intraoral (using a wrapped ice cube in the oral vestibule)^28^ and extraoral cold pack application^11,28^. Their results were not final, and the protocol was different in each but all are aligned in their findings that cold application decreases the level of POP.

Moreover, several investigations and meta-analyses have shown the effectiveness of cryotherapy in reducing pain following the surgical extraction of impacted mandibular third molars under local anesthesia^29–31^. These findings highlight the successful thermal transmission of cryotherapy through soft tissue and mandibular bone, supporting its efficacy as a pain management strategy in clinical practice.

As documented, cryotherapy’s efficacy is influenced by the thermal conductivity of the treated area^32^. Therefore, intraoral cryotherapy was chosen over extraoral application to mitigate the influence of muscles and lipids in the cheeks, which act as insulating barriers to deeper tissues^33^. Additionally, intraoral application allowed for a reduced application time of 30 min to minimize the risk of cryotherapy-related complications, such as nerve palsy^34^. Furthermore, to standardize the method of cold transmission, intraoral application was preferred over intracanal approaches, as a previous study noted variations in the degree of cold transmission to the periodontal ligament across different root sections during intracanal cryotherapy^12^. These variations were attributed to differences in dentine thickness and mineralization levels^12^.

The temperatures needed to reduce symptoms of the inflammatory response ranged from 10 to 15 °C within 15 min^35^. The temperature of the ice gel was measured to be 13.9° ± 4.1 °C^36^. However, because the temperature drops caused by cryotherapy vary widely depending on the body region, technique, and modality employed, an objective evaluation of tissue temperature (thermometer) was used to ensure that the required temperature is met throughout therapy^37^.

It seemed that there wasn’t much difference between the temperature reduction caused by ice and gel packs^38^. However, the gel pack was preferred due to its flexibility and ability to adapt to the oral vestibule^38^. In addition, when compared to an ice pack, both the ice and gel pack cooled the skin for 10 and 15 min, respectively. However, the gel pack maintained the lower temperature for a longer duration^37^.

There have been no clinical trials that have compared the effectiveness of intermittent and continuous cold therapy intraorally. In this study, an intermittent application of the gel pack was chosen. The pack was applied for 10 min, removed for 2 min, and then reapplied twice using a new pack each time to ensure temperature stability, which was monitored by a thermometer.

Following our root canal treatment, all patients in this research experienced considerable pain relief and did not require post-operative analgesics. This outcome lends credence to the findings that single-visit root canal therapy is an effective pain management technique for teeth with irreversible pulpitis^27^. The two groups did not differ in terms of gender, age, or preoperative pain demographics. Consequently, it is assumed that these factors did not affect the research findings.

According to our research, intraoral cryotherapy has a positive impact on reducing postoperative pain (POP) levels. POP levels decreased steadily during the monitoring period, which is consistent with previous studies^13–17^. For example, a study investigated the effectiveness of various cryotherapy treatments on reducing the incidence of POP in molars with symptomatic apical periodontitis and found that intraoral and extraoral cryotherapy treatments were just as effective as intracanal cryotherapy at reducing the incidence of POP^28^.

Contrary to our findings, a systematic review and meta-analysis, which had a limited number of studies as a drawback, indicated that intracanal cryotherapy may not significantly decrease post-endodontic pain^39^. Furthermore, in patients who have symptomatic irreversible pulpitis and normal periapical tissue, no significant difference in pain was observed between the cryotherapy and non-cryotherapy groups. However, this lack of significant difference may be due to the absence of inflammation in the periapical area among the cases included in the study^40^.

The physiological consequences of applying cold are closely tied to its beneficial effects. The first consequence is hemodynamic, whereby cold promotes vasoconstriction with an antiedema impact, resulting in a decrease in inflammation by reducing the number of leukocytes adhering to the capillary endothelial wall and minimizing their migration^41^. The second is cellular and has to do with slowing down metabolism; cells consume less oxygen after exposure to cold with its vasoconstriction effect which limits cellular damage^41^. The final one is neurological and linked with the effects on peripheral nerve endings since cold lowers the threshold for activating tissue nociceptors^41^ as well as the speed at which painful nerve impulses travel a phenomenon known as (cold-induced neurapraxia)^8^.

Recently, there has been a lot of interest in the role of nerves in controlling inflammatory processes through the production of neuropeptides^42^. Substance P is a neuropeptide that is produced by neurons in response to noxious stimuli and initiates inflammation^25,43^, thus, we assessed its level in this study. SP has considerable impacts on immunological reaction, and blood flow, playing a key role in inflammatory disorders like irreversible pulpitis^44,45^. All SP samples were collected by gathering apical fluids from the periapical area. This provides a significant advantage because it allows for the evaluation of the inflammatory mediators' response to all chemo-mechanical procedures in a closed human environment without any conflicts, as may be the situation with gingival crevicular fluid examination^46^. Bearing in mind that this study only included individuals who hadn’t used pre-operative analgesics in the seven days before therapy to eliminate any possible analgesic bias on SP expression^44^, our results of the elevated SP expression in the first sample are consistent with several studies confirming the presence of painfully inflamed pulps^22,47^. The study’s outcomes demonstrated that in teeth with symptomatic apical periodontitis, cryotherapy decreased the level of SP significantly in the apical fluid. Unfortunately, the denial of prior investigations concerning the impact of intraoral cryotherapy on substance P levels in apical fluid resulted in a lack of direct comparison. A previous study^47^ demonstrated a reduction in the quantity of the inflammatory mediator IL-6 in the periapical exudate, whereas another showed that IL-6 levels remained stable, but IL-8 levels reduced after cryotherapy^28^. The correlation between the amount of SP and POP was positive in this study which lined parallel to another research^48^. Additional research might be conducted to examine the effect of cryotherapy on neuropeptides and inflammatory mediators.

The following restrictions were confronted in this study: the results need to be validated by additional clinical studies with larger sample sizes because the level of SP in the periapical fluid showed great participant variability. Besides, the pain level was determined subjectively, and lastly, intraoral cryotherapy interfered with the ability of patients to become blind.

## Methods

### Study design and ethics declarations

**Figure 3:**
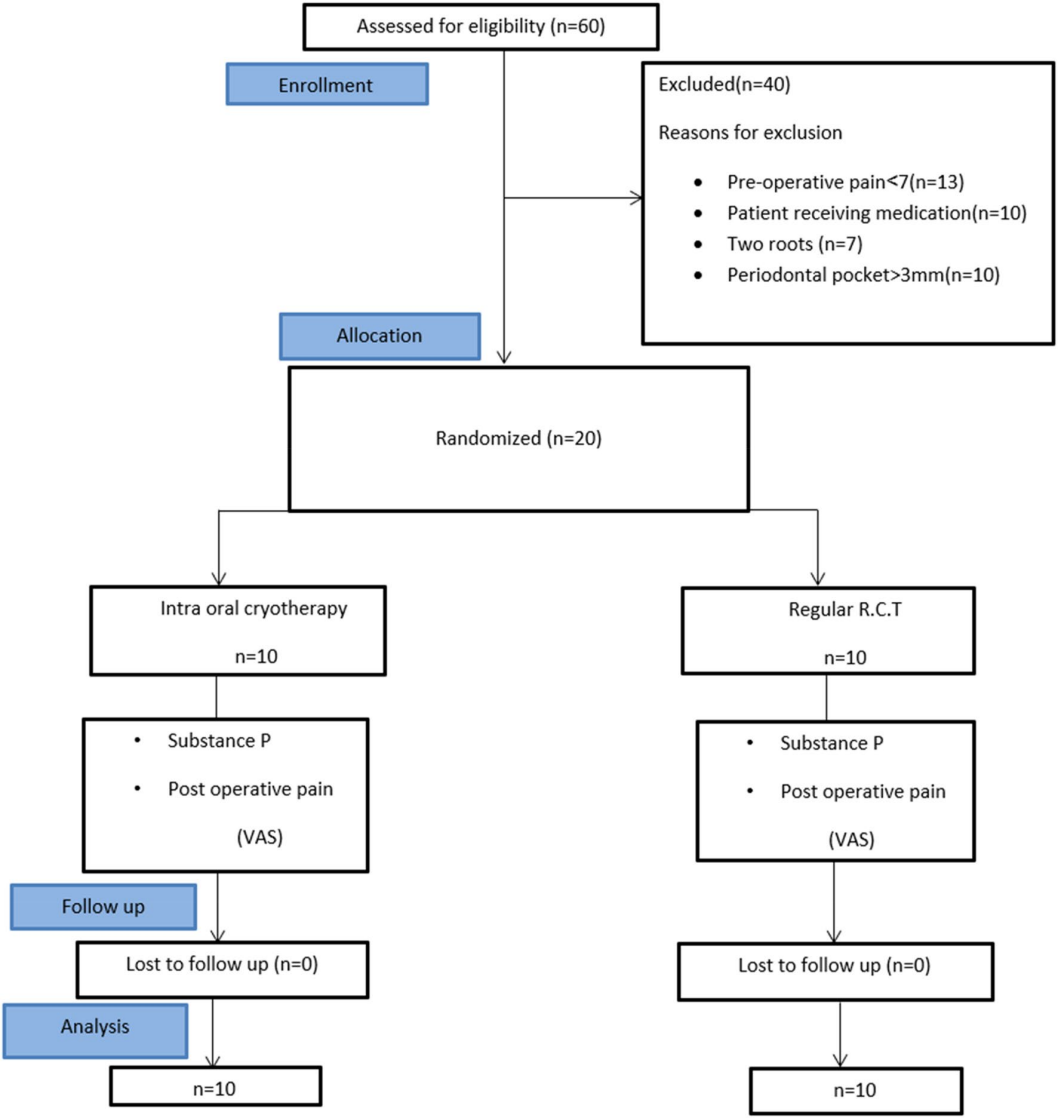
The consolidated standards of reporting trials flow diagram.

This was a randomized, controlled, single-center clinical trial that was planned and reported by the Consolidated Standards of Reporting Trials statement (CONSORT)^49^ and performed by the Declaration of Helsinki. Figure 3 is a flow diagram showing the study’s Consolidated Standards of Reporting Trials. It was assessed and then approved by the Ethics Committee, Faculty of Dentistry, Ain Shams University (FDASU) with Approval code: FDASU-RecIM012117. Then, it was registered in www.clinicaltrials.gov with an ID: NCT06082479 at (13/10/2023). All methodology in our research ran parallel to the guidelines and regulations of FDASU and informed consent was obtained from all participants included in the study.

The sample power analysis was carried out using data from a past study^28^. It shows that a total sample size of 10; subdivided into 5 in each group; will be adequate to detect effect size (t) = 2.3, with an actual power (1-β error) of 0.8 and a significance level (α error) of 0.05. To achieve 95%, the sample size was expanded to 20 (10 in each group).

### Patients’ selection

Patients referred to the Department of Endodontics with discomfort caused by irreversible pulpitis from carious teeth necessitating root canal therapy were considered potential research participants. All the information regarding the therapy and the research was illustrated adequately, and they were requested to sign an informed consent form. Eligible participants were between the ages of 18–45, had no systemic diseases, and had not taken any medication in the previous 7 days. Based on clinical symptoms of severe preoperative and percussive pain (VAS > 7), such patients should have a mandibular single-rooted premolar and Vertucci Type I canal system with signs of apical periodontitis. All teeth in the research reacted exaggeratedly to cold (Endo-Frost, Coltene-Whaledent, Switzerland) pulp sensibility tests, and copious bleeding of the pulp was visible upon getting access to the pulp chamber. Another criterion for study inclusion was the absence of any periapical bone abnormalities on preoperative periapical radiographs. Individuals who refused to sign an informed consent form or had any alteration in root canal anatomy were excluded from the study.

### Patients’ randomization, allocation, and preparation

Patients were assigned at random to either the Cryotherapy Group or the Control Group (n = 10). The group’s name was scrawled on a slip of paper and sealed inside a plain envelope. As each patient arrived for treatment, one envelope was drawn at random from a box to assign the patient to a certain group. To avoid operator bias, the closed envelopes containing information regarding the group participants assigned remained closed until the cold application phase.

In the beginning, the investigator HE assessed 60 patients; 13 had moderate preoperative pain (less than 7), 10 received medications during the previous week, 7 had double-rooted mandibular premolars discovered by preoperative radiograph and 10 patients had deep periodontal pockets. Thus, 40 patients were excluded and the remaining 20 met the inclusion criteria. Their demographic information (age and gender) was documented. The pain levels of each were recorded using a 10-point visual analogue scale (VAS) before treatment. The subjects reported their pain severity by selecting a number from 0 to 3 for mild pain, 4–6 for moderate pain, and 7–10 for severe pain.

Before the root canal treatment, inferior alveolar nerve block anesthesia (4% Artinibsa, 100,000 epinephrine) was administered. During treatment, each tooth was isolated with a rubber dam. The working length was adjusted to be 0.5 mm shorter than the entire length using an electronic apex finder, EPEX (Eighteenth Medical Technology Co., Ltd., Changzhou, China), and validated by a periapical radiograph. Root canal instrumentation began with a size 10 or 15 K-file (Mani, Co., Japan), and a glide path of up to 25 size K-file (Mani, Co., Japan) was made before rotary files were used. The canals were enlarged apically to a size 25 using a 6% taper rotary file (M PRO files (Guangdong, China, Mainland)) according to the manufacturer’s instructions. The canal was irrigated conventionally with 2 ml of 2.5% NaOCl between files. Finally, 5 ml of 2.5% NaOCl was applied for 1 min, followed by 5 ml of 17% EDTA (META BIOMED, Korea) for 1 min. Irrigants were delivered by a two-sided–vented needle, gauge size 25.

### Collection of Apical Fluid (AF) samples

The first AF samples (S1) were taken at the end of canal preparation. After confirming apical patency with a size 10 K-file, the paper point (size 20, 6% taper) (DiaDent Group, Seoul, Korea) was positioned 1–2 mm beyond the apex of the root canal and kept in place for 30 s to absorb enough fluid before being placed in an Eppendorf tube with 1 ml phosphate-buffered saline (PBS) (pH 7.4) and refrigerated at 10 °C^39^.

### Patients’ classification and interventions

Patients were randomly divided according to intraoral cold application into:

- **Cryotherapy group**: intraoral cryotherapy was done by placing a custom-made plastic pack (2.5 × 5 cm) containing ice gel (DonJoy Orthopaedic Pty Ltd, Normanhurst, New South Wales, Australia) intraorally on the buccal vestibule directly over the treated tooth. Patients were instructed to place the pack in their mouth for 10 min and to remove it for 2 min if they felt extremely cold or had a burning sensation^28^. During the cryotherapy treatment, a thermocouple device was used to monitor the temperature, which was maintained at 10 °C. The designated member of the research team (HE) ensured precise control and adherence to the prescribed temperature. If the temperature exceeded the predetermined threshold of 10 °C, immediate replacement of the gel pack with a new one was carried out to bring the temperature back within the desired range. Each pack was placed for 10 min, totaling three minimally per 30 min of intermittent application with 2-min breaks in between. After the completion of three cycles, the second sample of AF (S2) was collected, as described earlier.
- **Control Group**: the second sample of AF (S2) was collected 30 min after biomechanical preparation without applying any cold subject.

### Root canal obturation and POP level recordings

Root canal filling was performed on the same visit. Gutta-percha points (DiaDent Group, Seoul, Korea) and resin-based sealer (Adseal, Meta Biomed, Korea) were used to finish the root canal fillings. Before dismissal, patients were instructed to record their pain levels at 6, 24, 48, 72 h, and 7 days after treatment. The analgesic medication Cataflam 50 mg was prescribed to all patients, with instructions to take it every 8 h for 3 days if they experienced unbearable pain following the dental visit. The patients were tasked with recording their intake of the analgesic medication, as well as their pain levels.

### Biochemical examination

The paper points were separated and diluted in PBS (pH 7.4). The materials were then centrifuged at 3000 rpm for 10 min at 4 °C. Substance P was quantified using an enzyme-linked immunosorbent assay (ELISA) kit (no: E-EL-0067, Elabscience Biotechnology, China) according to the manufacturer’s instructions. With this kit, the Detection Range for SP was 78.13–5000 pg/ml. In a microplate reader, on a spectrophotometer (ELx 800; Bio-Tek Instruments Inc., Winooski, VT, USA), the absorbency of each sample was measured at 450 nm wavelengths. The standard concentrations of SP were used to produce a standard curve. The standard curve was used to calculate the concentration of SP in each sample.

### Statistical analysis

Data was gathered and analyzed statistically using IBM SPSS software version 23 (IBM Corp., USA). Fisher’s exact test was used to analyze categorical data that came in the form of frequency and percentage values. For numerical data, mean and standard deviation (SD) data were used. They were checked for normalcy using Shapiro–Wilk’s test and the data distribution. The independent t-test was used to analyze normally distributed data (age, substance P). Non-parametric data (VAS) were analyzed using the Mann–Whitney U test for intergroup comparisons and Friedman’s test for intragroup comparisons, followed by the Nemenyi Post Hoc test. Correlations were analyzed using Spearman’s rank-order correlation coefficient. The level of significance was fixed at P < 0.05.

## Data Availability

All data are available and can be presented upon request by contacting the corresponding author.
